# Periostin-mediated NOTCH1 activation between tumor cells and HSCs crosstalk promotes liver metastasis of small cell lung cancer

**DOI:** 10.1186/s13046-024-03266-7

**Published:** 2025-01-07

**Authors:** Linlin Lou, Keren Peng, Shumin Ouyang, Wen Ding, Jianshan Mo, Jiayu Yan, Xiaoxiao Gong, Guopin Liu, Jinjian Lu, Peibin Yue, Kai Zhang, Jian Zhang, Yan-dong Wang, Xiao-lei Zhang

**Affiliations:** 1 National-Local Joint Engineering Laboratory of Druggability and New Drug Evaluation, Guangdong Key Laboratory of Chiral Molecule and Drug Discovery, School of Pharmaceutical Sciences, Sun Yat-Sen University, Guangzhou, 510006 China; 2 State Key Laboratory of Ophthalmology, Zhongshan Ophthalmic Center, Sun Yat-Sen University, Guangzhou, 510060 China; 3 State Key Laboratory of Quality Research in Chinese Medicine, Institute of Chinese Medical Sciences, University of Macau, Macao, China; 4 Department of Medicine, Division of Hematology-Oncology, and Samuel Oschin Comprehensive Cancer Institute, Cedars-Sinai Medical Center, Los Angeles, CA 90048 USA; 5 Department of Thoracic Surgery, The Third Affiliated Hospital of Sun Yat-Sen University, Guangzhou, 510630 China

**Keywords:** Small cell lung cancer, Liver metastasis, Hepatic stellate cells, POSTN, Tumor microenvironment

## Abstract

**Background:**

Metastasis is the primary cause of mortality in small cell lung cancer (SCLC), with the liver being a predominant site for distal metastasis. Despite this clinical significance, mechanisms underlying the interaction between SCLC and liver microenvironment, fostering metastasis, remain unclear.

**Methods:**

SCLC patient tissue array, bioinformatics analysis were performed to demonstrate the role of periostin (POSTN) in SCLC progression, metastasis, and prognosis. Cell migration, invasion and sphere formation assay were performed to determine the oncogenic role of POSTN. RNA sequencing analysis was utilized to identify the key signaling pathway regulated by POSTN. Immunoprecipitation, immunofluorescence and co-culture system were used to clarify the mechanism of POSTN-NOTCH1 axis in tumor cells-hepatic stellate cells (HSCs) crosstalk. Subcutaneous xenograft model and liver metastasis model were established to examine the anti-tumor growth and metastases effect of targeting POSTN-NOTCH1 signaling axis.

**Results:**

Elevated expression of POSTN in SCLC is correlated with accelerated tumor progression and metastasis. Conditioned medium rich in POSTN derived from SCLC tumors demonstrates the ability to activate HSCs in the liver microenvironment. Mechanistically, POSTN emerges as a binding partner for the membrane receptor NOTCH1 and transducing the extracellular signals to intracellular fibroblasts. Furthermore, targeting the POSTN-NOTCH1 signaling axis proves effective in suppressing SCLC tumor growth and inhibiting liver metastasis. This study elucidates that the SCLC-derived secreted protein POSTN interacts with NOTCH1 on HSCs to promote the activation of HSCs, thereby providing a favorable microenvironment for liver metastasis.

**Conclusion:**

These findings uncover the intricate communications between primary SCLC cells and HSCs in the tumor microenvironment mediated by the secreted protein POSTN in the context of liver metastasis. Consequently, targeting the POSTN-NOTCH1 signaling axis emerges as a promising therapeutic strategy for metastatic SCLC.

**Supplementary Information:**

The online version contains supplementary material available at 10.1186/s13046-024-03266-7.

## Introduction

Lung cancer is the leading cause of cancer-related deaths globally, with small cell lung cancer (SCLC) accounting for approximately 15% of cases and characterized by rapid proliferation and high metastatic frequency [1, 2]. Metastases are found at initial diagnosis in approximately two-thirds of SCLC patients, despite advancements in therapy, resulting in an exceptionally poor prognosis, with only a 10% 5-year survival rate [3, 4]. During the past three decades, cisplatin–etoposide is still the first-line chemotherapy regimen [5, 6]. Other treatments, such as anti-PD1 antibody pembrolizumab plus chemotherapy demonstrate efficacy in extensive-stage SCLC patients but show no improvement in overall survival [7]. Metastasis is clinically the main cause of death in patients with SCLC. Given the aggressive signature of SCLC, diagnosis should be performed as early as possible. In addition to the usual imaging to define the extent of disease and blood tests, the development of circulating tumor cells (CTCs) and circulating cell-free DNA analysis is experimentally confirmed to evaluate SCLC heterogeneity [8, 9]. Liver metastasis, a prevalent occurrence in SCLC [10], significantly contributes to patient mortality, emphasizing the urgency for early diagnosis. Beyond conventional diagnostic approaches, exploration of the intricate mechanisms governing metastasis is imperative for timely intervention.

There are complicated communication mechanisms between the primary tumor and the distal metastasis [11]. Tumor cells may educate premetastatic niches of remote organs, recruit immune cells, and activate resident fibroblasts to support metastatic tumor microenvironment [12–15]. Fibroblasts are highly plastic and exhibit multi-potency, and can be reprogrammed into tumor-associated fibroblasts (CAFs) by tumor cells [16]. CAFs are the most abundant stromal cells in the tumor microenvironment and play an important role in tumor malignancy including angiogenesis, drug resistance, and metastasis [17–19]. Hepatic stellate cells (HSCs) are fibroblast-like pericytes, resident mesenchymal cells located in the perisinusoidal area between endothelial cells and hepatocytes and comprise between 5–10% of all resident cells within the liver. The activation and trans-differentiation of quiescent HSCs mainly trigger liver fibrosis and liver metastasis [20, 21]. Recent emerging evidence shows that SCLC secretes a variety of growth factors and cytokines to promote extracellular matrix (ECM) remodeling and angiogenesis [22]. However, it is unclear whether SCLC delivered specific secreted proteins involved in the functional remodeling of distant fibroblasts.

Periostin (POSTN) was first characterized from the periosteum and periodontal ligament in adult mice [23]. It encoded cysteine-enriched EMI domain, FAS1-repeated domain, and C-terminal variable domain [24]. The three domains have been found to interact with ECM protein including collagen, fibronectin, BMP-1, and Tenascin C, suggesting its contribution to ECM remodeling and cell migration [25–28]. Previous studies have indicated the potential involvement of POSTN in a variety of diseases such as inflammations, fibrosis, tumorigenesis, and metastasis [29–33]. Serum POSTN level is also aberrantly upregulated in lung cancer, and high POSTN level contributes to increasing T-stage and N-stage of patients with SCLC [34–36]. Some studies identify the involvement of POSTN in the several steps of the metastatic cascade. POSTN participates in tumor-proximal intravasation processes by elevating epithelial-mesenchymal transition (EMT) and motility of clear cell renal cell carcinoma [37]. Meanwhile, as a niche component in the latter steps of metastasis, POSTN promotes metastatic colonization of breast cancer stem cells by upregulating the Wnt pathway [11]. However, the contribution of POSTN amplification to SCLC metastasis is unclear. Specifically, the mechanism for how SCLC-derived POSTN affects tumor microenvironment has not been fully elucidated.

Herein, we systematically explore the oncogenic and metastatic role of POSTN in SCLC. High POSTN levels enhance SCLC tumor cell proliferation and metastatic dissemination. Our study reveals that SCLC-derived POSTN mediates collagen deposition and liver fibrosis, highlighting its crucial role in shaping the tumor microenvironment. Mechanistically, POSTN interacts with the NOTCH1 receptor, regulating the activation of HSCs through NOTCH1 signaling. This interaction facilitates crosstalk between SCLC cells and HSCs in the liver microenvironment, ultimately promoting liver colonization and metastasis. The study further suggests that targeting the POSTN/NOTCH1 axis may represent a novel strategy for managing SCLC liver metastasis.

## Results

### Role of POSTN in small cell lung cancer progression

**Fig. 1:**
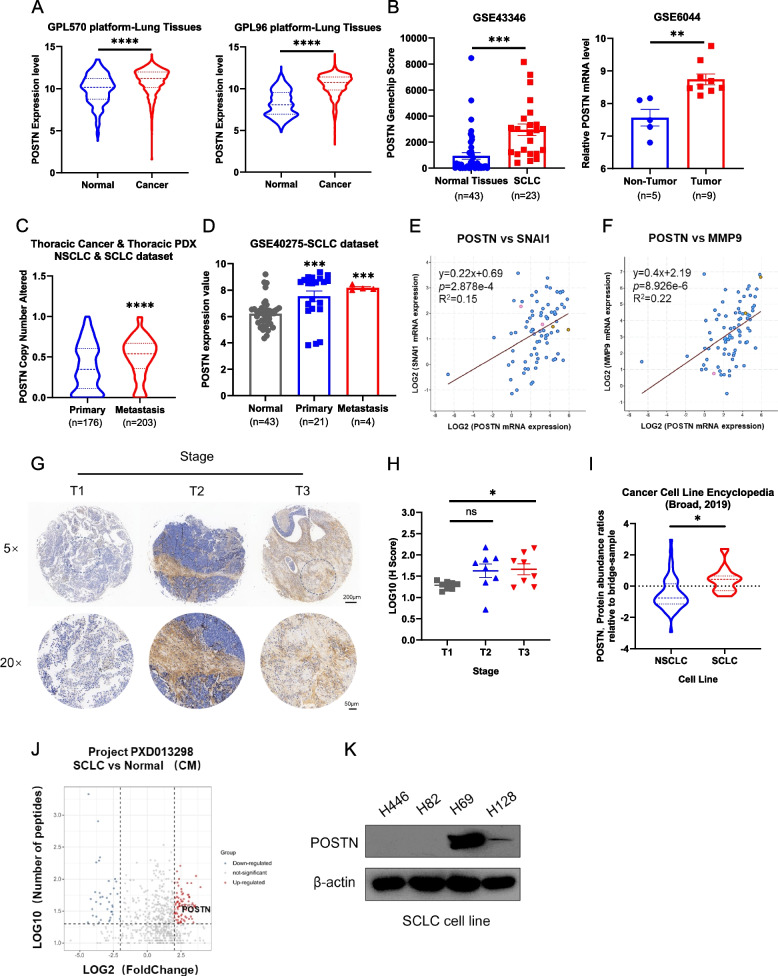
Secreted protein POSTN is aberrantly upregulated in small cell lung cancer. **A** POSTN expression levels in normal lung tissues and lung cancer tissues from GPL570 platform and GPL96 platform; **B** POSTN expression levels in various normal tissues (*n* = 40) and SCLC tissues (*n* = 23) in dataset GSE43346; POSTN levels in normal lung tissues (*n* = 5) and SCLC tissues (*n* = 9) in dataset GSE6044; **C** Altered copy numbers of POSTN in patients with primary lung cancers (*n* = 176) and metastatic lung cancers (*n* = 203) in Thoracic Cancer & Thoracic PDX datasets; **D** POSTN expression levels in normal lung tissues (*n* = 43), primary SCLC tissues (*n* = 21) and metastatic SCLC tissues (*n* = 4) in GSE40275 dataset; **E**–**F** Pearson’s correlation analysis of the expression levels of POSTN and WNT2/MMP2 in SCLC tumor samples from the cBioPortal database; **G** IHC images of POSTN in SCLC patient tissue array. Scale bars = 200 μm and 50 μm, respectively; **H** POSTN positive H-Score analyzed by AIpathwell software; **I** Relative protein levels of POSTN between NSCLC and SCLC cell lines from Cancer Cell Line Encyclopedia (Broad, 2019); **J** Volcano map of different proteins in conditioned medium (CM) between human SCLC cell lines and the human bronchial epithelial cell line in secretory proteomics of PXD013298; **K** Western blot analysis of POSTN protein levels in different SCLC cell lines. *P < 0.05, **P ≤ 0.01, ***P < 0.001, ****P ≤ 0.0001. *n* = 3. Student's t-test. All data were shown as means ± sem

To comprehensively understand the involvement of POSTN in small cell lung cancer (SCLC) progression, an initial exploration of the Gene Expression Omnibus (GEO) database was undertaken. The analysis revealed a significant upregulation of POSTN in SCLC as well as other lung cancer types when compared to normal tissues (Fig. 1A-B). Furthermore, POSTN expression exhibited a notable increase in metastatic lung cancer, particularly in metastatic SCLC (Fig. 1C-D). Intriguingly, POSTN demonstrated a marked elevation in liver metastatic tumors compared to primary tumors (Fig. S1A). Considering the pivotal role of matrix metalloproteinases (MMPs) and epithelial-to-mesenchymal transition (EMT) in the Wnt/β-catenin pathway, a pathway implicated in tumor metastasis [38], a correlation analysis was conducted using the cBioPortal database with 81 SCLC samples. POSTN can maintain stemness by stimulating Wnt signaling [11], and the analysis confirmed a positive correlation between POSTN and WNT2/MMP2 (Fig. 1E-F). Consistently, a similar positive correlation between POSTN and SNAI1/MMP9 was also observed (Fig. S1B-C). In addition, we further analyzed the co-expression between POSTN and genes related to the EMT pathway. The results showed a positive correlation between the expression of POSTN and LOX/TGFB1 genes, suggesting the involvement of POSTN in regulating the EMT process (Figure S1D-E). To further explore the potential oncogenic role of POSTN in SCLC, immunohistochemical (IHC) staining of SCLC patient tissue array was performed, revealing a high protein abundance of POSTN in stage III, indicative of a strong correlation between POSTN and SCLC malignancy (Fig. 1G-H) in the cohort (Supplementary Table S1). Subsequently, analysis of the Cancer Cell Line Encyclopedia database indicated higher relative protein levels of POSTN in SCLC cell lines compared to non-small cell lung cancer (NSCLC) cell lines (Fig. 1I). A quantitative secretome analysis by Yu et al. [39], provided additional evidence of POSTN enrichment in SCLC conditioned medium, supporting its specific association with SCLC progression (Fig. 1J). Further assessments of POSTN expression in various SCLC cell lines identified H128 and H69 as high-expression cell lines and H446 as a low-expression cell line for the following research (Fig. 1K). Taken together, these findings collectively underscore the oncogenic role of POSTN in SCLC.

### POSTN promotes the progression and metastasis of small cell lung cancer

**Fig. 2:**
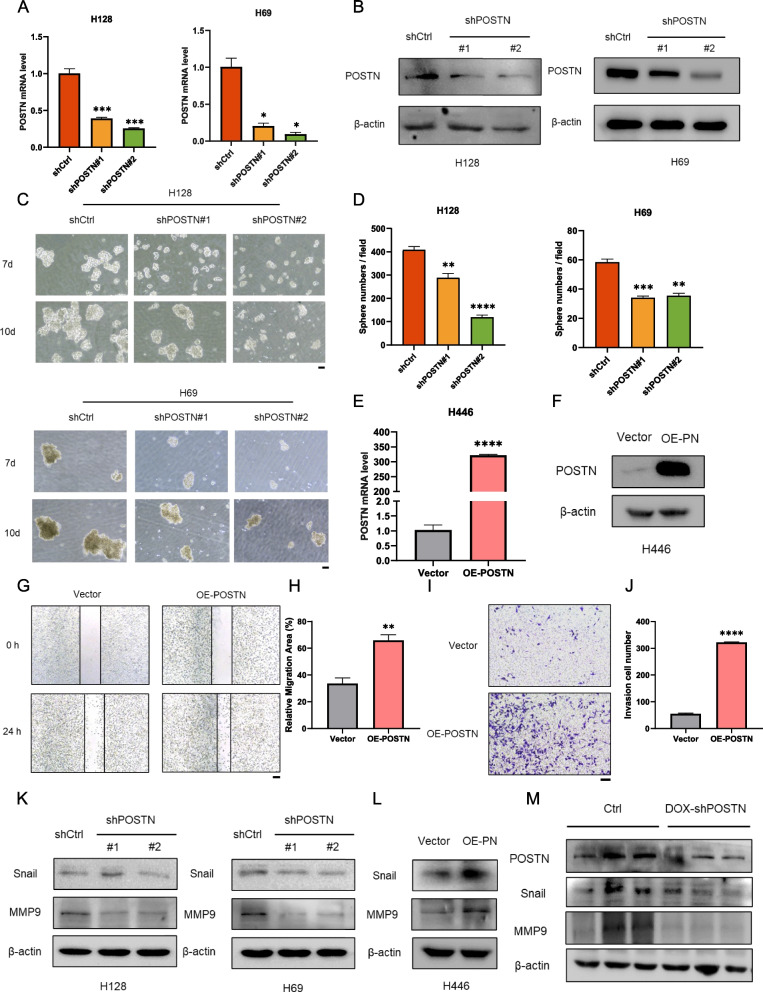
POSTN promotes the progression and metastasis of small cell lung cancer. **A**-**B** The mRNA levels (A) and protein levels (B) of POSTN in POSTN-knockdown H128 and H69 cells; **C**-**D** Sphere formation assay in POSTN-knockdown H128 and H69 cells (C), and the sphere numbers were quantified (D). Scale bar = 400 μm; **E**–**F** The mRNA levels (E) and protein levels (F) of POSTN in POSTN-overexpression H466 cells; **G**-**H** Cell migration in POSTN-overexpression H466 cells was detected by wound healing assay (G). Quantification of relative migration area was shown (H). Scale bar = 400 μm; **I**-**J **The invasion ability of POSTN-overexpression H466 cells was detected by transwell invasion assay (I). Quantification of invasion cell number was shown (J). Scale bar = 200 μm; **K** The protein levels of Snail and MMP9 in POSTN-knockdown H128 and H69 cells; **L** The protein levels of Snail and MMP9 in POSTN-overexpression H466 cells; **M** The protein levels of POSTN, Snail and MMP9 in mouse xenografts with inducible knockdown of POSTN. *P < 0.05, **P ≤ 0.01, ***P < 0.001, ****P ≤ 0.0001. *n* = 3. Student's t-test. All data were shown as means ± sem

The above observations link POSTN to the development and progression of small cell lung cancer, therefore, we hypothesized that POSTN plays a pivotal role in promoting the proliferation and metastasis of SCLC. To investigate the functions of POSTN, POSTN was stably knocked down in SCLC cell lines H128 and H69. The knockdown effect was confirmed by both mRNA and protein levels (Fig. 2A-B). Subsequent cell viability and count assays suggested that POSTN knockdown significantly inhibited the growth of SCLC cells (Fig. S2A-B). Notably, the downregulation of POSTN resulted in a diminished sphere-forming ability (Fig. 2C-D). Moreover, a stable POSTN-overexpressed cell line was established using H446 cells (Fig. 2E-F). As revealed by various functional assays in vitro, POSTN-overexpression enhanced the malignant properties of SCLC, encompassing enhanced cell proliferation, migration, and invasion (Fig. 2G-J and S2C-D). Furthermore, POSTN-knockdown markedly suppressed the expression of metastasis-related proteins such as Snail1 and MMP9, while POSTN overexpression promoted the upregulation of these metastatic biomarkers (Fig. 2K-L). To comprehensively assess the contribution of POSTN to SCLC progression in vivo, subcutaneous xenograft model utilizing doxycycline-inducible POSTN-knockdown H128 cells in BALB/c-nu/nu mice was established. Confirmatory qRT-PCR and western blotting validated the effective knockdown of POSTN after Doxycycline treatment in the xenograft mouse model (Fig. S2E-F). Inducing POSTN knockdown with Doxycycline treatment at a tumor size of 30 mm^2^ resulted in a significantly reduced tumor growth rate, evidenced by smaller tumor volumes and lighter tumor weights (Fig. S2G-I). Immunohistochemical (IHC) analysis of SCLC tumors verified the suppressive effects of POSTN-knockdown on Ki67 expression (Fig. S2J). Subsequent western blotting confirmed reduced expression of POSTN, Snail, and MMP9 in POSTN-knockdown xenograft tumor tissues, suggesting the inhibition of SCLC metastatic abilities through the downregulation of EMT-related genes (Fig. 2M). Taken together, these results demonstrate the pro-proliferative and pro-metastatic roles of POSTN in small cell lung cancer.

### Cancer-derived POSTN promotes the activation of hepatic stellate cells

**Fig. 3:**
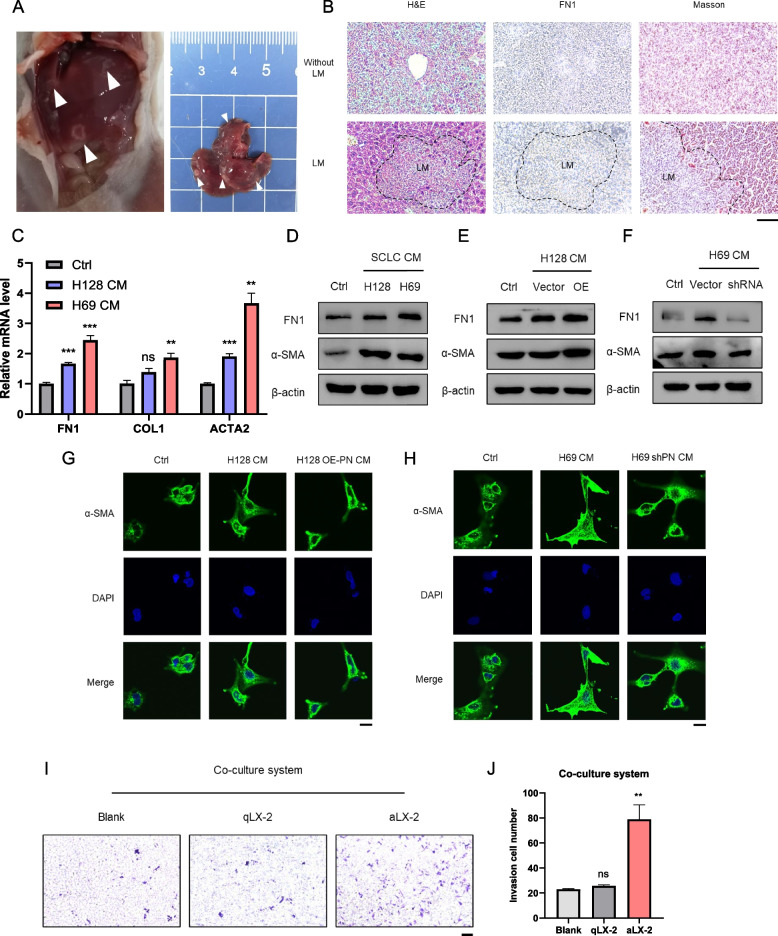
Cancer-derived POSTN affects the activation of hepatic stellate cells. **A** Representative tumor images of liver metastasis in SCLC subcutaneous graft model; **B** H&E, IHC, and Masson staining of liver without metastasis and liver with metastasis. Scale bar = 200 μm. LM stands for liver metastasis; **C** The mRNA levels of fibrosis markers in LX-2 cells treated with SCLC cells CM for 48 h; **D** The protein levels of fibrosis markers in LX-2 cells treated with SCLC cells CM for 48 h; **E**–**F** The protein levels of fibrosis markers in LX-2 cells treated with indicated SCLC cells CM for 48 h; **G**-**H** Immunofluorescence detection of α-SMA expression levels in LX-2 cells treated with indicated SCLC cells CM for 48 h. Scale bar = 10 μm. **I**-**J** The invasion ability of H466 cells co-cultured with qLX-2 and aLX-2 cells (quiescent, named qLX-2 or activated, named αLX-2) was detected by transwell invasion assay (I). Quantification of invasion cell number was shown (J). Scale bar = 200 μm. *P < 0.05, **P ≤ 0.01, ***P < 0.001, ****P ≤ 0.0001. *n* = 3. Student's t-test. All data were shown as means ± sem

Next, we sought to elucidate the role of POSTN in mediating crosstalk between SCLC and the liver microenvironment, both in cellular and murine models. The crucial biomarkers associated with liver metastasis were investigated. In the control group mice, visually detectable liver metastases were apparent, characterized by elevated Fibronectin 1 (FN1) expression and increased collagen deposition (Fig. 3A-B). This observation prompted our attention to the role of liver microenvironment in the formation of the liver pre-metastasis niche. Recognizing that hepatic stellate cell activation and collagen deposition are recurring events in tumor liver metastasis progression, we posed the question of whether POSTN-enriched conditioned medium (CM) plays a role in activating liver-resident fibroblasts. The LX-2 cell line, characterized as hepatic stellate cells (HSCs), are activated in response to stimuli such as inflammation. Activated HSCs promote liver cancer or liver metastases as cancer-associated fibroblasts (CAF)in the tumor microenvironment. We observed that LX-2 cells treated with POSTN-enriched CM resulted in a pro-fibrotic response characterized by the upregulation of fibrosis-related genes, including α-SMA and FN1 (Fig. 3C-D). Given that POSTN is reported to be secreted and carried in extracellular vehicles (EVs), we isolated and characterized EVs extracted from SCLC conditioned medium. Western blotting and nanoparticle tracking analysis confirmed the presence of POSTN-loaded EVs marked with specific indicators (CD63 and TSG101) and a size distribution peaking around 60 ~ 150 nm (Fig. S3A-B). Additionally, we investigated the impact of CM loaded with varying POSTN levels on LX-2. Application of CM derived from POSTN-overexpressed or -silenced SCLC cells to LX-2 for 48 h demonstrated that CM from POSTN-overexpressed SCLC cells promoted fibrosis in LX-2 cells, while POSTN-knockdown partially mitigated these effects (Fig. 3E-F). Consistently, immunofluorescence staining assays indicated correlations between POSTN and α-SMA, a key regulator of fibrosis (Fig. 3G-H and S3C-D).

Furthermore, activated hepatic stellate cells (aHSCs) have been implicated in the malignant phenotype of hepatocellular carcinoma (HCC) through paracrine signaling. To assess whether aHSCs contribute to the invasion ability of SCLC cells, a co-culture system was established for invasion assays. SCLC cells and aHSCs were applied in the upper and lower compartments of the transwell chambers, respectively. The results demonstrated that aHSCs can enhance the invasion ability of SCLC cells (Fig. 3I-J). These results underscore the potential interplay between tumor cells, hepatic stellate cells, and the liver microenvironment in fostering the progression of small cell lung cancer.

### Enriched conditioned medium containing POSTN activated HSCs via NOTCH signaling axis 

**Fig. 4:**
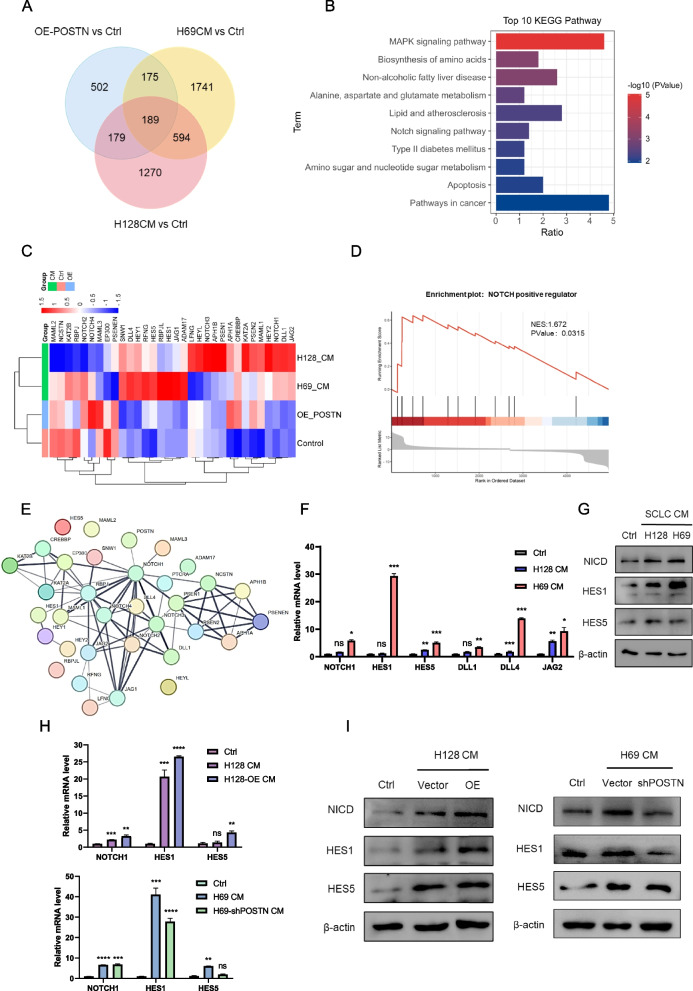
POSTN-enriched conditioned medium activated HSCs via NOTCH signaling axis. **A** Venn map of up-regulated genes in SCLC CM treated group and OE-POSTN group; **B** KEGG pathway enrichment analysis of significantly up-regulated genes in both SCLC CM group; **C** Cluster heat map of positive regulators in NOTCH pathway; **D** GSEA enrichment analysis of NOTCH pathway positive regulators in LX-2 cells treated with H69 CM; **E** The String analysis of the interaction between POSTN and NOTCH positive regulators; **F** The mRNA levels of indicated genes in LX-2 cells treated with SCLC CM for 48 h; **G** The indicated protein levels in LX-2 cells treated with SCLC CM for 48 h; **H** The mRNA levels of indicated genes in LX-2 cells treated with different SCLC CM for 48 h; **I** The indicated protein levels in LX-2 cells treated with different SCLC CM for 48 h. *P < 0.05, **P ≤ 0.01, ***P < 0.001, ****P ≤ 0.0001. *n* = 3. Student's t-test. All data were shown as means ± sem

To elucidate the molecular mediators governing the interaction between POSTN-positive (POSTN +) small cell lung cancer (SCLC) cells and HSCs, we conducted RNA-seq transcriptomic profiling and subsequent gene enrichment analysis. This analysis revealed 189 significantly up-regulated genes (fold change > 2) in both SCLC conditioned medium (CM) treated groups and POSTN-overexpression (OE-POSTN) groups (Fig. 4A). KEGG pathway enrichment analysis further highlighted prominent pathways, including the MAPK signaling pathway, Non-alcoholic fatty liver disease (NAFLD), and the Notch signaling pathway in SCLC CM treated groups (Fig. 4B). Considering the implication of the NOTCH signaling pathway in HSC activation and liver fibrosis [40, 41], we examined the transcriptomic profiles of NOTCH positive regulators in various treatment groups. A cluster heatmap confirmed that SCLC CM treatment upregulated the expression of NOTCH1 and several canonical ligands and downstream genes, including DLL1/4, JAG2, target genes HES1/5, HEY2, and transcription factor RBPJL (Fig. 4C). Gene-set enrichment analysis (GSEA) further demonstrated that POSTN-loaded CM treatment exhibited enrichment of a gene expression signature corresponding to NOTCH positive regulators (Fig. 4D). Given that secreted proteins can influence the intracellular fates of membrane receptors [42, 43], we further investigated the protein–protein interaction between POSTN and NOTCH positive regulators using string network analysis. The results identified NOTCH1 as a potential binding partner of POSTN (Fig. 4E).

Subsequently, we explored the impact of POSTN on NOTCH1 activation. qRT-PCR and Western blot analyses confirmed that SCLC CM treatment and POSTN-expression induced the activation of NOTCH1 signaling in LX-2 cells, as evidenced by the upregulation of NOTCH1, HES1/5, DLL1/4, and JAG2 (Fig. 4F-G and S4A-B). Moreover, POSTN was enriched in CM from POSTN-overexpressed H128 cells, and POSTN-knockdown in H69 cells abrogated the protein level in CM. As demonstrated in Fig. 4H-I, POSTN-enriched CMs elevated the protein expression levels of NICD and HES family, while POSTN silencing produced opposing effects. Collectively, these results suggest that POSTN-enriched CM may facilitate the activation of HSCs, which is mediated by the NOTCH1 signaling axis through paracrine loops.

### POSTN directly binds to membrane receptor NOTCH1 to promote the activation of hepatic stellate cells

**Fig. 5:**
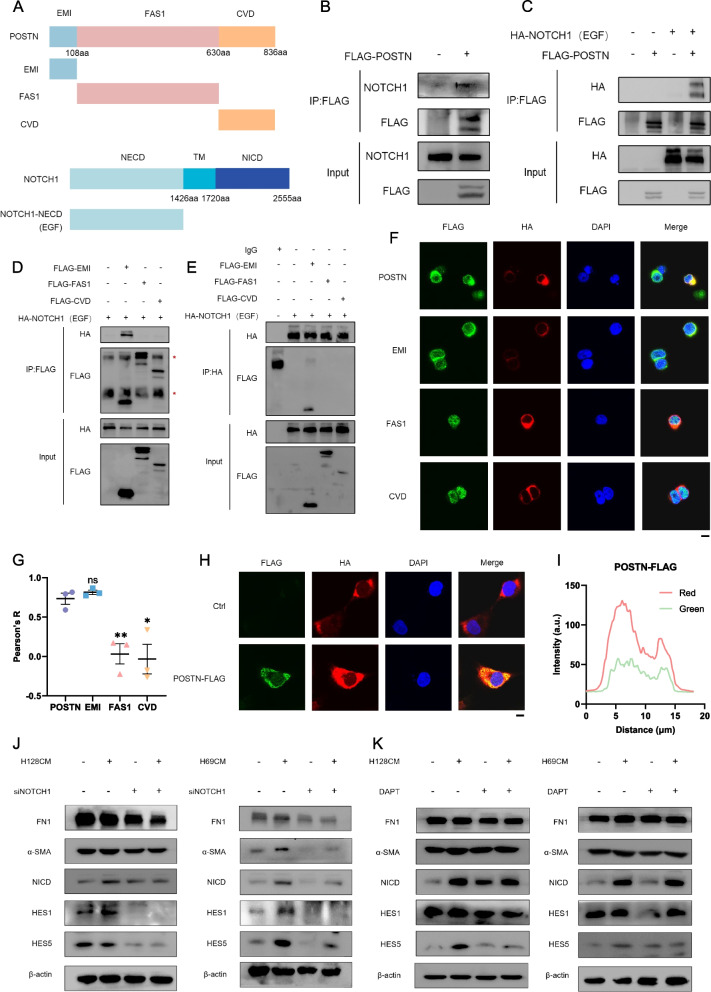
POSTN directly binds to membrane receptor NOTCH1 to promote the activation of hepatic stellate cells. **A** Schematic diagram of POSTN and NOTCH1 truncations; **B**-**C** Co-IP was used to analyze the interaction between POSTN and full-length NOTCH1 (B) or NOTCH1-NECD domain (C) in 293T cells; **D** Pull down FLAG to analyze the interaction between NOTCH1-NECD domain and different POSTN truncations in 293T cells; **E** Pull down HA to analyze the interaction between NOTCH1-NECD domain and different POSTN truncations in 293T cells; **F**-**G** The co-localization of POSTN full-length or truncations and NOTCH1-NECD domain in 293T cells detected by Immunofluorescence analysis (F). Quantification of the Pearson value was shown (G). Scale bar = 10 μm; **H**-**I**. **I** The co-localization of exogenous POSTN and NOTCH1-NECD domains in LX-2 cells treated with CM from POSTN-FLAG-overexpression H446 cells detected by Immunofluorescence analysis (H). Quantification of the fluorescence intensity was shown (I). Scale bar = 10 μm; **J** The indicated proteins level in LX-2 cells treated with SCLC CM and/or siNOTCH as indicated for 48 h; **K** The indicated proteins level in LX-2 cells treated with SCLC CM and/or DAPT (20 μM) as indicated for 48 h. *P < 0.05, **P ≤ 0.01, ***P < 0.001, ****P ≤ 0.0001. *n* = 3. Student's t-test. All data were shown as means ± sem

To elucidate the underlying mechanism how the tumor cells derived POSTN promote fibrosis in HSCs, we identified potential binding partners through string analysis. Consequently, POSTN and NOTCH1 truncations, containing crucial domains, were cloned for subsequent experiments (Fig. 5A). Co-immunoprecipitation (Co-IP) and immunoblot experiments in 293T cells confirmed NOTCH1, particularly its NECD domain, is a binding partner of POSTN (Fig. 5B-C). Further investigation into the specific domains involved in this interaction revealed that the NECD domain primarily interacts with the EMI domain of POSTN, as evidenced by Co-IP and immunofluorescence (IF) assays (Fig. 5D-E). IF staining indicated a notable co-localization between the NECD domain and the EMI domain, with a higher average co-localization index (Fig. 5F-G and S5A). Additionally, conditioned medium from H446 cells with overexpression of POSTN-FLAG induced membrane co-localization of the NECD domain in LX-2 cells (Fig. 5H-I). These findings collectively suggest that the secreted protein POSTN from SCLC interacts with the membrane receptor NOTCH1, transmitting extracellular signals to the intracellular LX-2.

To delineate the downstream pathway through which POSTN affects LX-2 activation, alterations in POSTN content in SCLC CM, interference with NOTCH1 expression, and inhibition of NOTCH1 functional activation were investigated. Genetic inhibition of NOTCH1 significantly alleviated the activation of the NOTCH1 signaling pathway, leading to downregulation of fibrotic markers, including FN1 and α-SMA, in response to SCLC CM-induced HSC activation (Fig. 5J). Similar alleviative effects were observed in POSTN-overexpression-activated HSCs (Fig. S4C). Pharmacological inhibition of NOTCH1 signaling using the γ-secretase inhibitor DAPT further validated fibrosis suppression via inhibition of the NOTCH1 axis (Fig. 5K). Notably, DAPT treatment demonstrated safety profiles on LX-2 cells, as TGFb-induced fibrosis, rather than proliferation, was selectively abrogated (Fig. S5B-D). These results underscore the activation of fibroblasts through the POSTN-NOTCH1 signaling pathway and provide insight into the mechanism by which POSTN regulate hepatic microenvironment.

### Targeting the POSTN-NOTCH1 signaling axis suppresses SCLC tumor growth *in vivo*

**Fig. 6:**
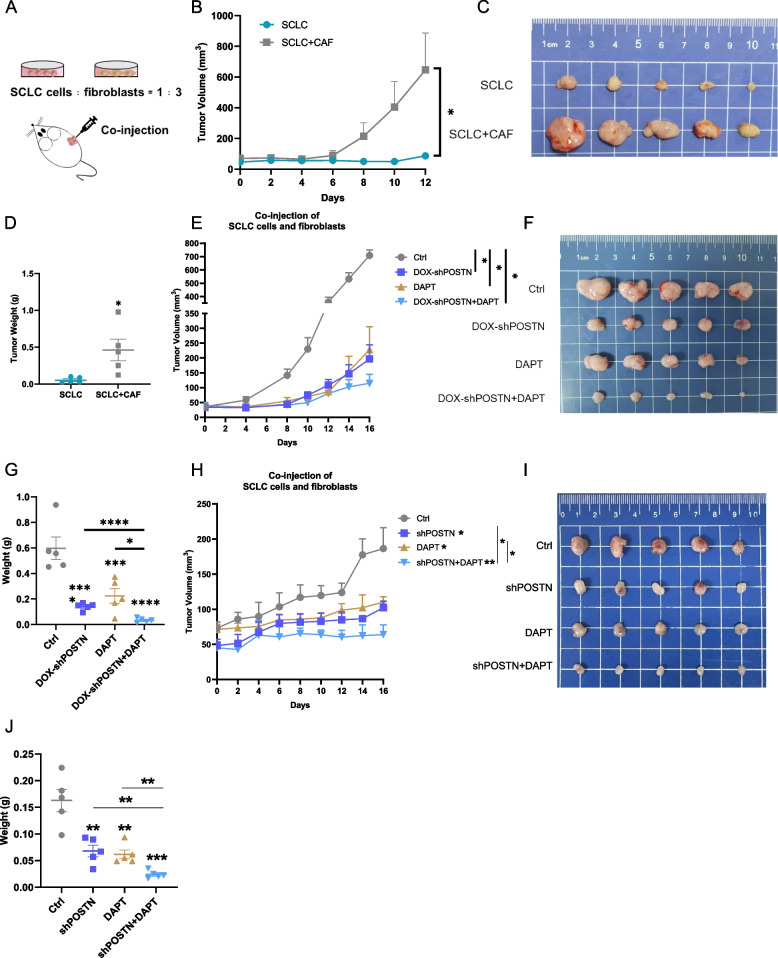
Targeting the POSTN-NOTCH1 signaling axis suppresses SCLC tumor growth in vivo*.* **A** Experimental schematic illustrating the establishment of co-injection model and the indicated drug treatment; **B** Tumor size from mice injected with SCLC cells and HSCs in 12 days; **C** Representative subcutaneous tumor images on day 12; **D** Statistical chart of the tumor weight in day 12; **E** Tumor growth curve of mice injected with H128 cells and LX-2 cells in 16-day drug administration; **F** The appearance of the subcutaneous tumor after 16 days of drug administration; **G** The subcutaneous tumor weight after 16-day drug administration. **H** Tumor growth curve of mice injected with H69 cells and LX-2 cells in 16-day drug administration; **I** The appearance of the subcutaneous tumor after 16 days of drug administration; **J** The subcutaneous tumor weight after 16-day drug administration. *P < 0.05, **P ≤ 0.01, ***P < 0.001, ****P ≤ 0.0001. *n* = 3. Difference between two groups was tested by two-sided Student’s t test; the analyses of experiments with multiple groups were performed by one-way analysis of variance (ANOVA). All data were shown as means ± sem

To assess the impact of the POSTN-NOTCH1 axis on SCLC tumor growth, we established a subcutaneous xenograft model in BALB/c-nu/nu mice by co-injecting SCLC cells (H128) and HSCs (LX-2), in a 1:3 ratio (Fig. 6A). The results showed that HSCs significantly promoted SCLC tumor growth in the co-injection group, leading to a substantial increase in tumor size and final tumor weight (Fig. 6B-D). This observation underscores the pivotal role of CAFs in supporting and enhancing SCLC tumor growth.

To delve deeper into the contribution of the POSTN-NOTCH1 axis to the tumor microenvironmental "interactome", co-injected mice were administered with vehicle, doxycycline water, NOTCH1 inhibitor DAPT, and a combination of both, respectively. The results suggested that POSTN knockdown in SCLC was essential for the anticancer effects in our co-injection model, with limited therapeutic efficacy observed upon NOTCH1 blockade alone. Notably, the combined inhibition of POSTN in SCLC and the NOTCH pathway in HSCs synergistically inhibited SCLC proliferation, resulting in a significant reduction in tumor growth (Fig. 6E-G). Similar synergistic effects were observed in the H69 cells and HSCs co-injection xenograft model. POSTN knockdown in combination of NOTCH inhibitor DAPT led to synergistic tumor growth regression compared with monotherapy (Fig. 6H-I). These findings highlight the potential therapeutic benefit of simultaneously targeting the POSTN-NOTCH1 axis in both cancer cells and the tumor microenvironment for effective suppression of SCLC tumor growth in vivo.

### Targeting the POSTN-NOTCH1 signaling axis inhibits SCLC liver metastasis *in vivo*

**Fig. 7:**
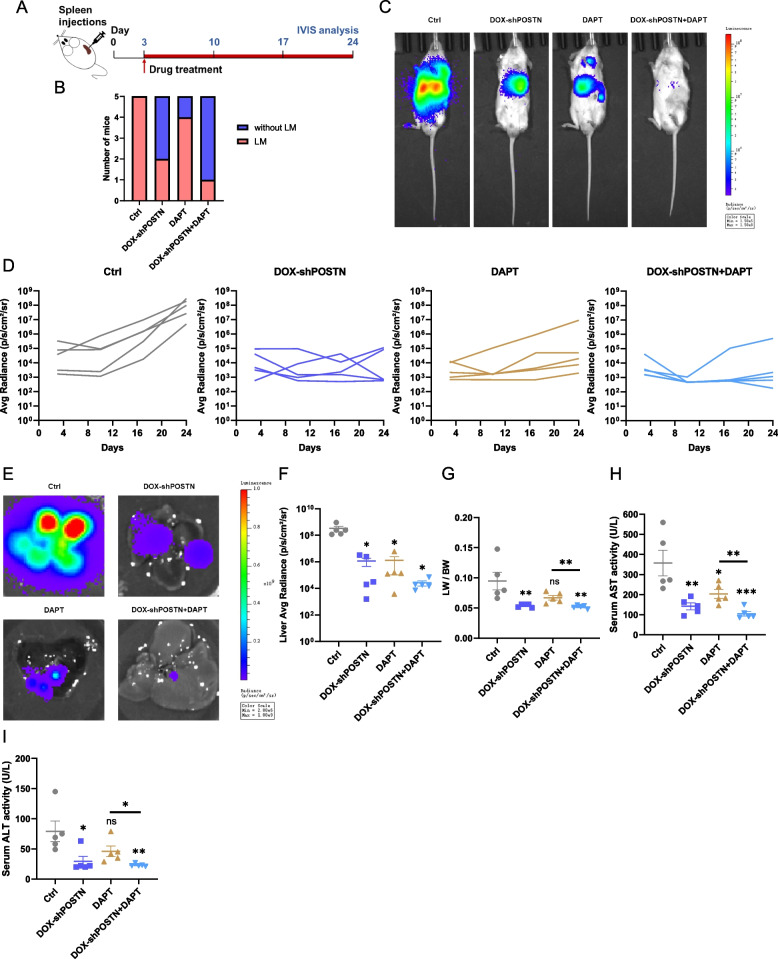
Targeting the POSTN-NOTCH1 signaling axis inhibits SCLC liver metastasis in vivo*. ***A** Experimental schematic illustrating the establishment of liver metastasis model and the indicated drug treatment; **B** Representative bioluminescence images of liver metastasis models on day 24; **C** Number of mice with or without liver metastasis (LM); **D** Quantification of photon flux in 24 days; **E**–**F** Representative bioluminescence images of livers on day 24 (E). Quantification of liver average radiance was shown (F); **G** Quantification of liver weight (LW) / body weight (BW) ratios on day 24. **H**-**I** Serum AST activity (H) and ALT activity (I) of mice on day 24. *P < 0.05, **P ≤ 0.01, ***P < 0.001, ****P ≤ 0.0001. *n* = 3. Difference between two groups was tested by two-sided Student’s t test; the analyses of experiments with multiple groups were performed by one-way analysis of variance (ANOVA). All data were shown as means ± sem

To further assess the impact of the POSTN-NOTCH1 axis on SCLC liver metastasis, we established spleen injection mouse models to specifically induce liver metastasis. Four distinct treatment regimens, including vehicle, doxycycline water, NOTCH1 inhibitor DAPT, and their combination, were administered to the mice (Fig. 7A). Emphasizing liver metastasis over other metastatic sites, we observed consistent results in the spleen injection mouse models, where SCLC depletion via POSTN knockdown significantly reduced the incidence of liver metastasis. Importantly, the combined therapy exhibited remarkable efficacy in inhibiting the occurrence of liver metastasis in mice (Fig. 7B).

To investigate the localization of SCLC in the liver during metastasis, we harvested the livers and observed a significant reduction in bioluminescence signals in the drug administration groups on day 24. Consistent with the overall bioluminescence signals from the body, the combination of POSTN knockdown and NOTCH1 inhibitor DAPT notably suppressed bioluminescence signals in liver areas (Fig. 7C-D). Subsequent experiments were conducted to assess global liver function. The mice in combination therapy group exhibited a lower tumor burden than the mice for control, as indicated by lower liver-to-body weight ratios (LW/BW) (Fig. 7G). Aspartate aminotransferase (AST) and alanine aminotransferase (ALT) are clinically biomarkers of hepatocellular injury and disease [44]. The results confirmed that the combined therapy not only reduced the risk of liver injury but also improved hepatocellular function compared to DAPT monotherapy (Fig. 7H-I). Collectively, data from the liver metastasis models underscore the efficacy of targeting the POSTN-NOTCH1 signaling axis in suppressing the liver localization of SCLC.

## Discussion

In this study, we present compelling evidence supporting the role of the secreted protein POSTN as an oncogene in the progression of small cell lung cancer (SCLC). Our findings underscore that POSTN amplification facilitates tumor metastasis through intricate crosstalk between SCLC cancer cells and HSCs.

SCLC poses a significant global health challenge due to its dismal prognosis and limited treatment options. Typically diagnosed at an advanced, metastatic stage, SCLC necessitates novel diagnostic and therapeutic approaches. Liquid biopsies, especially those examining secreted proteins, emerge as promising tools for noninvasive early detection in SCLC patients [9]. As the important analytes from various biological fluids, secreted proteins mediate molecular communications between tumor cells and their adjacent microenvironment, which has been shown to be an important mechanism for promoting tumor growth, invasion, or angiogenesis. Secreted proteins can be secreted extracellularly into blood or body fluids, so they may be potential biomarkers for rapid, noninvasive monitoring of SCLC early detection [45]. Our study provides comprehensive evidence supporting the pivotal role of POSTN in driving SCLC progression and metastasis. Integrated analyses of the GEO database, cBioPortal, and SCLC tissues reveal POSTN as a potential driver of SCLC progression, correlating positively with malignant phenotypes both in vitro and in vivo. Known for its involvement in cell adhesion and motility, POSTN's downstream pathways, including integrin/FAK/Src and Wnt/β-catenin, are implicated in metastasis. Our data confirm that POSTN promotes metastasis by inducing epithelial-mesenchymal transition (EMT), particularly by upregulating MMP9 and Snail.

The observed liver metastasis in our xenograft mouse model underscores the significance of POSTN enrichment in SCLC metastasis. During liver metastasis, HSCs are activated, leading to extracellular matrix (ECM) deposition, providing a favorable environment conducive to cancer cell migration and angiogenesis. POSTN has been reported to be a positive regulator of HSC activation [46, 47]. SCLC is a highly heterogeneous malignant tumor, and the interaction between tumor cells and non-tumor cells in SCLC remains unclear. Our preliminary exploration into the interaction between SCLC cells and distal fibroblasts reveals that SCLC affects the activation of liver fibroblasts through secreted proteins, with POSTN playing a crucial signaling role.

The NOTCH receptor, a transmembrane protein with extracellular (NECD), transmembrane (TM), and intracellular (NICD) domains, was reported to be associated with fibrosis. NOTCH precursor protein goes through a series of cleavage events, including gamma-secretase, and further releases activated form NICD which enters the nucleus and initiates the transcription of downstream genes. Activation of the NOTCH pathway triggers liver fibrosis, as confirmed in nonalcoholic steatohepatitis (NASH) mouse models [48]. NOTCH antagonists and gamma-secretase inhibitors (GSI) effectively reduce hepatic fibrosis in patients with NASH [40–48]. RNA-seq analysis suggests that NOTCH1 is a downstream target of POSTN. Our proposed mechanism involves fibroblast reprogramming mediated by POSTN-dependent crosstalk between SCLC cells and the liver metastasis microenvironment, facilitated by the POSTN-NOTCH1 axis. The EMI domain of POSTN is implicated in the membrane co-localization of NOTCH1 receptor. SCLC-delivered POSTN signals communicate with fibroblasts, resulting in high NICD levels and liver fibrosis, reversible by NOTCH1 interference and γ-secretase inhibitor treatment. Notably, LX-2 cells exhibit insensitivity to γ-secretase inhibitor treatment, suggesting alternative growth signaling pathways.

Beyond cancer development, fibroblasts, abundant in the tumor stroma, contribute significantly to tumor recurrence and drug resistance, impacting treatment outcomes. [49]. Thus, targeting activated fibroblasts emerges as a potential avenue for improving therapeutic efficacy. Our study substantiates the proliferative role of the cancer-supporting niche through in vivo co-culture systems. Employing Dox-inducible POSTN knockdown in SCLC and NOTCH1 functional inhibitor DAPT in fibroblasts effectively improved the anti-tumor efficacy, offering a novel therapeutic strategy to impede SCLC liver metastasis. Importantly, our experimental SCLC models shed light on the intricacies of the cancer niche upon tumor cells reaching distant organs, providing valuable insights for targeted strategies in the liver microenvironment.

## Conclusion

**Fig. 8:**
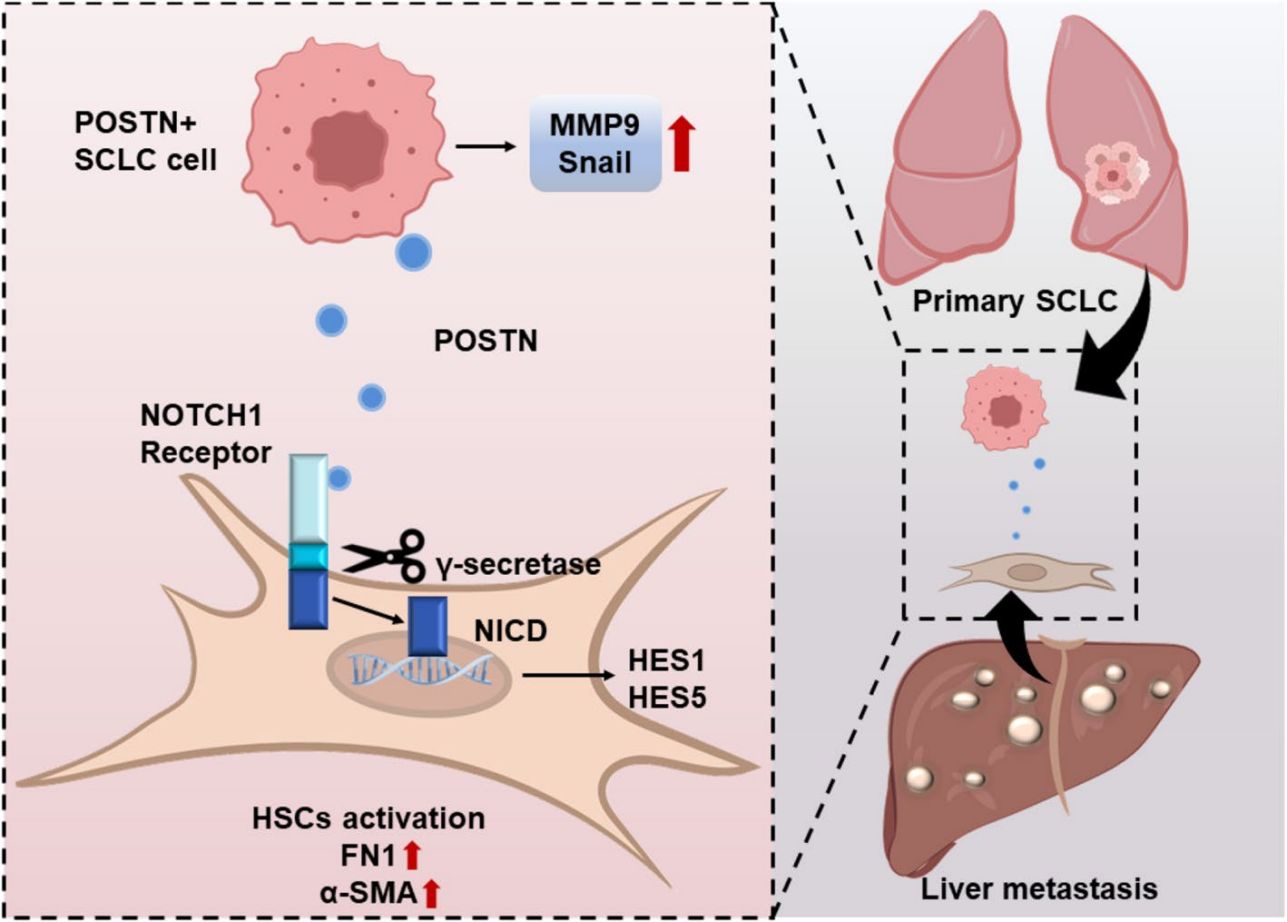
Summary illustration

In summary, our results unveil POSTN as a key driver in SCLC growth and metastasis, contributing to a fibrosis-supportive phenotype in the tumor niche. SCLC-derived secreted protein POSTN interacts with NOTCH1 on HSCs to promote the activation of HSCs, thereby providing a favorable microenvironment for liver metastasis. Significantly, targeting POSTN enhances the effectiveness of DAPT monotherapy. This finding suggests that targeting the POSTN-NOTCH axis might be a promising therapeutic strategy to curb SCLC progression and liver metastasis, offering potential insights into the development of novel treatment modalities for this aggressive disease (Fig. 8).

## Materials and methods

### Cell lines and human tissues

HEK293T cell line, human SCLC cell lines H128, H69, H446, H82 and human hepatic stellate cell line LX-2 were obtained from American Type Culture Collection (ATCC). HEK293T were cultured in DMEM medium supplemented with 10% FBS and 1% penicillin/streptomycin. H128, H69, H446, H82, and LX-2 were cultured in RPMI1640 medium supplemented with 10% FBS and 1% penicillin and streptomycin. All cells were cultured in 37 ℃ incubator containing 5% CO_2_. Lung cancer tissues were purchased from Xi’an bioaitech Co., Ltd (Xi’an, China). The samples were analyzed by IHC staining using anti-POSTN antibody according to the standard method.

### EVs purification and characterization

When cells reached about 80% confluence, the supernatants of cells were collected. Extracellular vesicles (EVs) were extracted by sequential centrifugation steps. The collected supernatants were centrifuged at 10,000 × g for 30 min and 110,000 × g for 70 min. The pellets were then washed with PBS and diluted with 50 μL PBS. The size of the purified vesicles was characterized by DynaPro Plate Reader II (WYATT technology, USA). Western blot was used to determine the positive staining of exosome markers CD63 and TSG101 after detecting the protein concentration.

### Construction of the expression vectors

Human *POSTN* gene (NM_006475) and truncations were amplified by reverse transcription polymerase chain reaction (RT-PCR) and inserted into the expression vector pEB-3 × flag-Puro. They were eventually named pEB-POSTN-3 × flag-Puro, pEB-EMI-3 × flag-Puro, pEB-FAS1-3 × flag-Puro, and pEB-CVD-3 × flag-Puro, respectively. The pLV3-CMV-NOTCH1-NECD (1-1426aa)-HA-Puro plasmid was constructed by MiaoLing Biology. To clarify the confirmation of successful insertion of nucleotide sequences of POSTN into expression vectors, we listed our sequencing results of POSTN in supplementary file.

POSTN-Forward Primer:5’-CGGCTAGCATGATTCCCTTTTTACCCATG-3’.

POSTN-Reverse Primer:5’-CCGCTCGAGCTGAGAACGACCTTCCCTTAA-3’.

EMI-Forward Primer:5’-CGGCTAGCATGATTCCCTTTTTACCCATGTTTT-3’.

EMI-Reverse Primer:5’-CCGCTCGAGCACGATGCCCAGAGTGCC-3’.

FAS1-Forward Primer:5’-CGGCTAGCATGGGAGCCACCACAACGCAG-3’.

FAS1-Reverse Primer:5’-CCGCTCGAGTGGATAGAGGAGTTTATCTACAACATGA-3’.

CVD-Forward Primer:5’-CGGCTAGCATGGCAGACACACCTGTTGGAAA-3’.

CVD-Reverse Primer:5’-CCGCTCGAGCTGAGAACGACCTTCCCTTAA-3’.

The shRNA sequences targeting POSTN were obtained from Millipore Sigma and are synthesized by Sangon Biotech (Shanghai, China). Annealed oligos were ligated into pLKO.1 vector or Tet-pLKO-Hygro vector according to Addgene protocols [50].

shPOSTN#1:

Forward Primer: 5’-CCGGCGGTGACAGTATAACAGTAAACTCGAGTTTACTGTTATACTGTCACCGTTTTTG-3’.

Reverse Primer: 5’-AATTCAAAAACGGTGACAGTATAACAGTAAACTCGAGTTTACTGTTATACTGTCACCG-3’.

shPOSTN#2:

Forward Primer: 5’-CCGGCACTTGTAAGAACTGGTATAACTCGAGTTATACCAGTTCTTACAAGTGTTTTTG-3’.

Reverse Primer: 5’-AATTCAAAAACACTTGTAAGAACTGGTATAACTCGAGTTATACCAGTTCTTACAAGTG-3’.

DOX-shPOSTN:

Forward Primer: 5’-CTAGCCGGTGACAGTATAACAGTAAATACTAGTTTTACTGTTATACTGTCACCGTTTTTG-3’.

Reverse Primer: 5’-AATTCAAAAACGGTGACAGTATAACAGTAAAACTAGTATTTACTGTTATACTGTCACCGG-3’.

### RNA interference

Briefly, hepatic stellate cells with 80% confluence were transiently transfected with siNOTCH1 or control siRNA using DharmaFECT (T-2001–03, Dharmacon, USA) according to the manufacturer’s instructions. After 6-h transfection, the cells were cultured in suitable medium for another 48–72 h. The following siRNA sequences for targeting genes were synthesized by Sangon Biotech (Shanghai, China).

siNOTCH1:

sense: 5'-CCCUUUGAGUCUUCAUACA-3';

antisense: 5'-UGUAUGAAGACUCAAAGGG-3'

### Cell transfection and lentivirus infection

HEK293T cells were seeded into 6-well plates and cultured until about 80% confluence. Then, cells were transfected with pLKO.1 vector and the packaging plasmid psPAX2 and pMD2.G, using the Lipofectamine 2000 kit (Invitrogen) according to the manufacturer’s instructions. The transfecting fluid was replaced with DMEM medium supplemented with 10% FBS after 10–12 h incubation. After another 48 h, the recombinant lentivirus was filtered with 0.22 μm membrane (Merck Millipore) and collected.

For lentivirus infection, the cells were infected by virus together with 8 μg/mL polybrene (Sigma), and screened by 2.5 μg/mL puromycin (MCE). The stable puromycin-resistant cells were collected for subsequent experiments.

### EdU assay

The proliferation of POSTN-overexpressed H446 cells was detected by using EdU kit according to the manufacturers’ instructions. Briefly, 8 × 10^4^ H446 cells were seeded into 12-well plates and cultured overnight. Then, cells were labeled by 1 × EdU working solution and incubated for 2 h. After EdU labeling, cells were fixed with 4% paraformaldehyde and permeabilized with 0.3%Triton X-100. The cells was dyed using click reaction solution and captured under a fluorescence microscope. DAPI was used to stain the cell nuclei. Three random fields of each section were captured with EVOS FL Auto Cell Imaging System (Life Technologies, USA).

### Cell migration and invasion assays

For cell migration assay, 1–1.5 × 10^6^ H446 cells were seeded into 6-well plates and cultured overnight. Then, scratches were made in the confluent monolayers using a pipette tip, followed by washing the wounds twice with PBS. Images of cells migrating to the wound area at both 0 and 24 h were captured using a phase-contrast microscope (Nikon). Finally, the distance migrated by the cells was measured by using ImageJ software.

The cell invasion assay was performed in 24-well Transwell® plates (Costar, USA). 6 × 10^4^ H446 cells were seeded in the top chamber of the insert with RPIM-1640 culture medium containing 2% FBS, while the bottom chamber was added with 500 μL of RPIM-1640 medium containing 20% FBS. The assay was performed after cultured in 37 ℃ incubator containing 5% CO2 for 24 h. Then, the migrated cells were stained and counted using crystal violet. Three random fields of each section were captured with Nikon microscopy.

### Sphere formation assay

The sphere formation assay was performed in Ultra-Low Attachment 6-Well Plates (Crystalgen, Ninbo). 3 × 10^4^ SCLC cells were seeded and clutured with DMEM-F12 medium containing 0.4% BSA, 5 μg/mL insulin, 20 ng/mL EGF, 20 ng/mL FGF, and 1% P.S. The random fields of each well were captured with Nikon microscopy.

### RNA sequencing and bioinformatics analysis

Total RNA was extracted by TRizol (YEASEN, China) from LX-2 cells, transfected with POSTN-overexpressed vector, and treated with H128 CM or H69 CM for 48 h and RNase-free DNase I to remove genomic DNA contamination. RNA integrity was evaluated with a 1.0% agarose gel. Thereafter, the quality and quantity of RNA were assessed using a NanoPhotometer® spectrophotometer (IMPLEN, CA, USA). The high-quality RNA samples were subsequently submitted to the Sangon Biotech (Shanghai, China) for library preparation and sequencing. Sequencing libraries were generated using VAHTSTM mRNA-seq V2 Library Prep Kit for Illumina® following the manufacturer's recommendations and index codes were added to attribute sequences to each sample. The libraries were then quantified and pooled. Paired-end sequencing of the library was performed on the HiSeq XTen sequencers (Illumina, San Diego, CA). FastQC (version 0.11.2) was used for evaluating the quality of sequenced data. Clean reads were mapped to the reference genome by HISAT2 (version 2.0) with default parameters. RSeQC (version 2.6.1) was used to analyze the alignment results. Gene expression values of the transcripts were computed by StringTie (version 1.3.3b). Library preparation and high-throughput sequencing were performed by Sangon Biotech (Shanghai, China).

### Quantitative real-time PCR

Total RNA was extracted using the Trizol reagent (YEASEN, China). The cDNA was synthesized using HifairTM II 1st Strand cDNA Synthesis SuperMix Kit (YEASEN, China) according to the manufacturer’s protocol. Detection on cDNAs was performed using SYBR Green (11201ES08*, YEASEN, China) with the required primers (Supplementary Table 2). The data were normalized to the expression of β-actin by utilizing the standard 2 − ΔΔCT method. All the primers were synthesized by Sangon Biotech (Shanghai, China).

### Western blot and antibodies

Cells or tumor tissues were harvested and lysed in RIPA buffer (Beyotime, Shanghai, China) supplemented with protease inhibitors (Beyotime, Shanghai, China) and phosphatase inhibitors (Bimake, USA). The supernatant was collected after centrifugation and protein concentration was evaluated by BCA Protein Assay Kit (23,225, Thermo, USA). 20–50 μg of proteins per sample were loaded and separated by SDS polyacrylamide gel. Subsequently, proteins were transferred to polyvinylidene fluoride membranes (PVDF, IPVH00010, Millipore, USA) and blocked with 5% non-fat milk in TBST. The blocked membranes were incubated with primary antibodies overnight at 4℃ (Supplementary Table 3). Appropriate goat anti-mouse or goat anti-rabbit secondary antibodies were used and the immunoblots were recorded with the Bio-rad chemidoc MP system after incubating with ECL solution.

### Immunofluorescence staining

1–1.5 × 10^5^ cells were seeded into 15 mm glass-bottom dishes and cultured overnight. Then, cells were fixed with 4% paraformaldehyde and permeabilized with 0.3%Triton X-100. Goat serum (Boster, Wuhan, Hubei, China) was used to block the samples for 30 min at room temperature. After that, the cells were incubated with Flag-tag and HA-tag primary antibody overnight at 4℃. The samples were rinsed three times in PBS for 5 min each. Next, cells were incubated with Alexa Fluor 555 Conjugate and Alexa Fluor 647 Conjugate for 1 h at room temperature in the dark. DAPI was used to stain the cell nuclei. Images were captured by Laser Scanning Confocal Microscope FV3000 (Olympus, Japan).

### Tumor mouse models

All the mouse experiments were reviewed and approved by the Research Ethics Committee of Sun Yat-sen University (SYSU-IACUC- 2022003356, SYSU-IACUC- 2023000557). BALB/c-nu/nu mice (male, age 4–6 weeks, SPF grade) and NOD-SCID (male, age 4–6 weeks, SPF grade) were purchased from the Experimental Animal Center of Sun Yat-sen University (Guangdong, China).

For the subcutaneous transplantation tumor models, 1 × 10^6^ briefly, H128-DOX-shPOSTN cells were suspended in a total of 100 μL PBS and matrigel (1:1, v/v) and injected into the subcutaneous area of Balb/c-nu/nu mice. The tumor volume was evaluated using a standard caliper and calculated using the formula: V = Length × Width^2^/2. When the tumor volume reached around 50mm^3^, mice were randomly divided into two groups (*n* = 7): Vehicle or Doxycycline. The Dox-shPOSTN group was administered by drinking water dissolved doxycycline at 2 mg/mL concentration in 2% sucrose solution.

For co-injection experiments, H128 cells alone (1 × 10^6^ cells per 100 μL PBS) or together with LX-2 cells (3 × 10^6^ cells per 100 μL PBS; ratio, 1:3) were suspended in a total of 100 μL PBS and matrigel (1:1, v/v) and injected into the subcutaneous area of Balb/c-nu/nu mice. Co-injection mice were randomly divided into four groups and administrated vehicle, DOX supplement, γ-secretase inhibitor DAPT (20 mg/kg, 3 times per week) or combined therapy. Mice were sacrificed and their tumors were harvested, immersed in 4% paraformaldehyde for paraffin sections, which were then used for histological examination.

NOD-SCID (male, age 4–6 weeks, SPF grade) were used for liver metastasis experiments. The experimental liver metastasis model was constructed by intra-splenic injection of a Luc-labeled H128-DOX-shPOSTN single-cell suspension at 3 × 10^6^ cells per 25 μL PBS. Living images were captured with IVIS Lumina II (PerkinElmer). 24 days later, mice were sacrificed, and their livers were harvested for bioluminescence signals analysis.

### Immunohistochemistry (IHC) staining

Paraffin-embedded tissue slides were deparaffinated for 45 min at 60 ℃. Then, rehydration was proceeded with following steps, three washes of xylene for 15 min each, two washes of 100% ethanol for 5 min each, one wash of 95% ethanol and 85% ethanol for 5 min, and three times in ddH_2_O for 1 min each. The slides were heated in 1 × sodium citrate buffer using a microwave and went through the progression of boiling, sub-boiling, and room temperature. After washing once in PBS, 3% hydrogen peroxide was used to incubate slides for another 10 min. Then, the slides were blocked with goat serum and incubated overnight with primary antibodies. Next, IHC staining was performed with HRP-conjugated secondary antibodies and the signal was detected by applying DAB staining. Nuclei were counterstained with Hematoxylin, following dehydration in an ethanol series and mounting. Images were captured with Nikon microscopy.

### Masson’s trichrome, and Sirius red staining

The slides were deparaffinized and rehydrated using a standard protocol. For Masson’s trichrome staining, Weigert’s Iron hematoxylin solution was used to stain the cell nuclei. The liver tissue slides were then stained in Ponceau-Acid Fuchsin Solution for 10 min and Aniline Blue Solution for 2 min, respectively. Finally, the slides were dehydrated in an ethanol series rapidly and mounted for examination. Images were captured with Nikon microscopy.

For Sirius Red staining, Iron Hematoxylin Staining Solution was used to stain the cell nuclei. Sirius Red Staining Solution was used to dye collagen fibers for 10 min following by dehydrating and mounting. Images were captured with Nikon microscopy.

### Determination of serum ALT and AST levels

Retro-orbital blood was collected for serum ALT and AST activity assay at the Guangdong Engineering & Technology Research Center for Disease-Model Animals at SUN Yat-sen University.

## Statistical analysis

All the experiments were performed three times and data were presented as mean ± standard error of the mean (SEM), unless otherwise stated. Statistical analysis was performed using GraphPad Prism 8.0 software (GraphPad Software, San Diego, CA). Difference between two groups was examined by two-sided Student’s t test; the analyses of experiments involving multiple groups were performed by one-way analysis of variance (ANOVA). *P* < 0.05 was considered to be a statistically significant difference.

## Supplementary Information

Supplementary Material 1.

Supplementary Material 2.

## Data Availability

The data and material that support the findings of this study are available on request from the corresponding author. The data are not publicly available due to privacy or ethical restrictions.

## Abbreviations

SCLC: Small cell lung cancer
CAFs: Cancer associated fibroblasts
POSTN: Periostin
CM: Conditioned medium
EMT: Endothelial-mesenchymal transition
ECM: Extracellular matrix
HSCs: Hepatic stellate cells
MMPs: Matrix metalloproteinases
IHC: Immunohistochemical
DOX: Doxycycline
FN1: Fibronectin 1
EVs: Extracellular vesicles
NAFLD: Non-alcoholic fatty liver disease
IF: Immune fluorescence
AST: Aspartate aminotransferase
ALT: Alanine aminotransferase
TGI: Tumor growth inhibition
GSEA: Gene set enrichment analysis
α-SMA: Alpha-smooth muscle action
