# Supplementary material for: Periostin-mediated NOTCH1 activation between tumor cells and HSCs crosstalk promotes liver metastasis of small cell lung cancer
Source: J Exp Clin Cancer Res. 2025 Jan 7;44:6. doi: 10.1186/s13046-024-03266-7 (PMC11706058; doi:10.1186/s13046-024-03266-7)
Supplement: Supplementary file 1 — Supplementary Material 1. [file 13046_2024_3266_MOESM1_ESM.docx]

**Sequencing result** (The highlighted texts are the inserted target gene sequence.)

**POSTN**

**#1**

GGAGGGGGGCTGATTATTCCTTCATGTATAACAATTTGGGGCTTTTCATTAACCATTATCCTAATGGGGTTGTCACTGTTAATTGTGCTCGAATCATCCATGGGAACCAGATTGCAACAAATGGTGTTGTCCATGTCATTGACCGTGTGCTTACACAAATTGGTACCTCAATTCAAGACTTCATTGAAGCAGAAGATGACCTTTCATCTTTTAGAGCAGCTGCCATCACATCGGACATATTGGAGGCCCTTGGAAGAGACGGTCACTTCACACTCTTTGCTCCCACCAATGAGGCTTTTGAGAAACTTCCACGAGGTGTCCTAGAAAGGATCATGGGAGACAAAGTGGCTTCCGAAGCTCTTATGAAGTACCACATCTTAAATACTCTCCAGTGTTCTGAGTCTATTATGGGAGGAGCAGTCTTTGAGACGCTGGAAGGAAATACAATTGAGATAGGATGTGACGGTGACAGTATAACAGTAAATGGAATCAAAATGGTGAACAAAAAGGATATTGTGACAAATAATGGTGTGATCCATTTGATTGATCAGGTCCTAATTCCTGATTCTGCCAAACAAGTTATTGAGCTGGCTGGAAAACAGCAAACCACCTTCACGGATCTTGTGGCCCAATTAGGCTTGGCATCTGCTCTGAGGCCAGATGGAGAATACACTTTGCTGGCACCTGTGAATAATGCATTTTCTGATGATACTCTCAGCATGGATCAGCGCCTCCTTAAATTAATTCTGCAGAATCACATATTGAAAGTAAAAGTTGGCCTTAATGAGCTTTACAACGGGCAAATACTGGAAACCATCGGAGGCAAACAGCTCAGAGTCTTCGTATATCGTACAGCTGTCTGCATTGAAAATTCATGCATGGAGAAAGGGAGTAAGCAAGGGAGAAACGGTGCGATTCACATATTCCGCGAGATCATCAAGCCAGCAGAGAAATCCCTCCATGAAAAGTAAAACAAGATAAGCG

**#2**

TCCCCTATCCGGATCTTGTGATCCAGTTAGGGTTGAATTCTGCTCTGAGGCGGATGGAGAATACACTTTGCTGGCCCTGTGAATAATGCATTTTCTGATGATACTCTCAGCATGGATCAGCGCCTCCTTAAATTAATTCTGCAGAATCACATATTGAAAGTAAAAGTTGGCCTTAATGAGCTTTACAACGGGCAAATACTGGAAACCATCGGAGGCAAACAGCTCAGAGTCTTCGTATATCGTACAGCTGTCTGCATTGAAAATTCATGCATGGAGAAAGGGAGTAAGCAAGGGAGAAACGGTGCGATTCACATATTCCGCGAGATCATCAAGCCAGCAGAGAAATCCCTCCATGAAAAGTTAAAACAAGATAAGCGCTTTAGCACCTTCCTCAGCCTACTTGAAGCTGCAGACTTGAAAGAGCTCCTGACACAACCTGGAGACTGGACATTATTTGTGCCAACCAATGATGCTTTTAAGGGAATGACTAGTGAAGAAAAAGAAATTCTGATACGGGACAAAAATGCTCTTCAAAACATCATTCTTTATCACCTGACACCAGGAGTTTTCATTGGAAAAGGATTTGAACCTGGTGTTACTAACATTTTAAAGACCACACAAGGAAGCAAAATCTTTCTGAAAGAAGTAAATGATACACTTCTGGTGAATGAATTGAAATCAAAAGAATCTGACATCATGACAACAAATGGTGTAATTCATGTTGTAGATAAACTCCTCTATCCAGCAGACACACCTGTTGGAAATGATCAACTGCTGGAAATACTTAATAAATTAATCAAATACATCCAAATTAAAGTTTGTTCGTATAGCACCTTTCAAGAAATCCCCGTGACTGTCTATAAGCCAATTTATTTAAAAAAAAAAATAC

**#3**

GGGCGCCAGAATGATACCTTCTGGTGATGAATTGAAATCAAAAGAATCTGACATCATGACAACAAATGGTGTAATTCATGTTGTAGATAAACTCCTCTATCCAGCAGACACACCTGTTGGAAATGATCAACTGCTGGAAATACTTAATAAATTAATCAAATACATCCAAATTAAGTTTGTTCGTGGTAGCACCTTCAAAGAAATCCCCGTGACTGTCTATAAGCCAATTATTAAAAAATACACCAAAATCATTGATGGAGTGCCTGTGGAAATAACTGAAAAAGAGACACGAGAAGAACGAATCATTACAGGTCCTGAAATAAAATACACTAGGATTTCTACTGGAGGTGGAGAAACAGAAGAAACTCTGAAGAAATTGTTACAAGAAGACACACCCGTGAGGAAGTTGCAAGCCAACAAAAAAGTTCAAGGATCTAGAAGACGATTAAGGGAAGGTCGTTCTCAGCTCGAGGAAGTCGACACTCCCGGGGGATCCATGGACTACAAAGACCATGACGGTGATTATAAAGATCATGACATCGATTACAAGGATGACGATGACAAGTGAGCGGCCGCGAAGGATCTGCGATCGCTCCGGTGCCCGTCAGTGGGCAGAGCGCACATCGCCCACAGTCCCCGAGAAGTTGGGGGGAGGGGTCGGCAATTGAACGGGTGCCTAGAGAAGGTGGCGCGGGGTAAACTGGGAAAGTGATGTCGTGTACTGGCTCCGCCTTTTTCCCGAGGGTGGGGGAGAACCGTATATAAGTGCAGTAGTCGCCGTGAACGTTCTTTTTCGCAACGGGTTTGCCGCCAGAACACAGCTGAAGCTTCGAGGGGCTCGCATCTCTCCTTCACGCGCCCGCCGCCCTACCTGAGGCCGCCATCCACGCCGGTTGAGTCGCGTTCTGCCGCCTCCCGCCTGTGGTGCCTCCTGAACTGCGTCCGCCGTCTAGGTAAGTTAAAGCTCAGGTCGAGACCGGGCCTTTGTCCGGGGTTCCTTGGAGCTACCAAAATCAG
