# Supplementary material for: Periostin-mediated NOTCH1 activation between tumor cells and HSCs crosstalk promotes liver metastasis of small cell lung cancer
Source: J Exp Clin Cancer Res. 2025 Jan 7;44:6. doi: 10.1186/s13046-024-03266-7 (PMC11706058; doi:10.1186/s13046-024-03266-7)
Supplement: Supplementary file 2 — Supplementary Material 2. [file 13046_2024_3266_MOESM2_ESM.docx]

**Supplementary materials for**

**Periostin-mediated NOTCH1 activation between tumor cells and HSCs crosstalk promotes liver metastasis of small cell lung cancer**

Linlin Lou#^1^, Keren Peng#^1^, Shumin Ouyang#^1^, Wen Ding^1,2^, Jianshan Mo^1^, Jiayu Yan^1^, Xiaoxiao Gong^1^, Guopin Liu^1,2^, Jinjian Lu^3^, Peibin Yue^4^, Kai Zhang^5^, Jian Zhang^5*^, Yan-dong Wang^2*^, Xiao-lei Zhang^1*^

1. National-Local Joint Engineering Laboratory of Druggability and New Drug Evaluation, Guangdong Key Laboratory of Chiral Molecule and Drug Discovery, School of Pharmaceutical Sciences, Sun Yat-sen University, Guangzhou 510006, China
2. State Key Laboratory of Ophthalmology, Zhongshan Ophthalmic Center, Sun Yat-sen University, Guangzhou 510060, China
3. State Key Laboratory of Quality Research in Chinese Medicine, Institute of Chinese Medical Sciences, University of Macau, Macao, China
4. Department of Medicine, Division of Hematology-Oncology, and Samuel Oschin Comprehensive Cancer Institute, Cedars-Sinai Medical Center, Los Angeles, CA, 90048, USA
5. Innovation Practice Center, Changchun University of Chinese Medicine, Changchun, 130117, China
6. Department of Thoracic Surgery, The Third Affiliated Hospital of Sun Yat-sen University, Guangzhou, China, 510630

#These authors contributed equally to this work.

*To whom correspondence should be addressed:

E-mail: zhangxlei5@mail.sysu.edu.cn (Xiaolei Zhang), wangydsyj@163.com (Yan-dong Wang), sumszhangjian@163.com (Jian Zhang)

**Supplemental Figure1-5**

**Supplemental Table1-2**


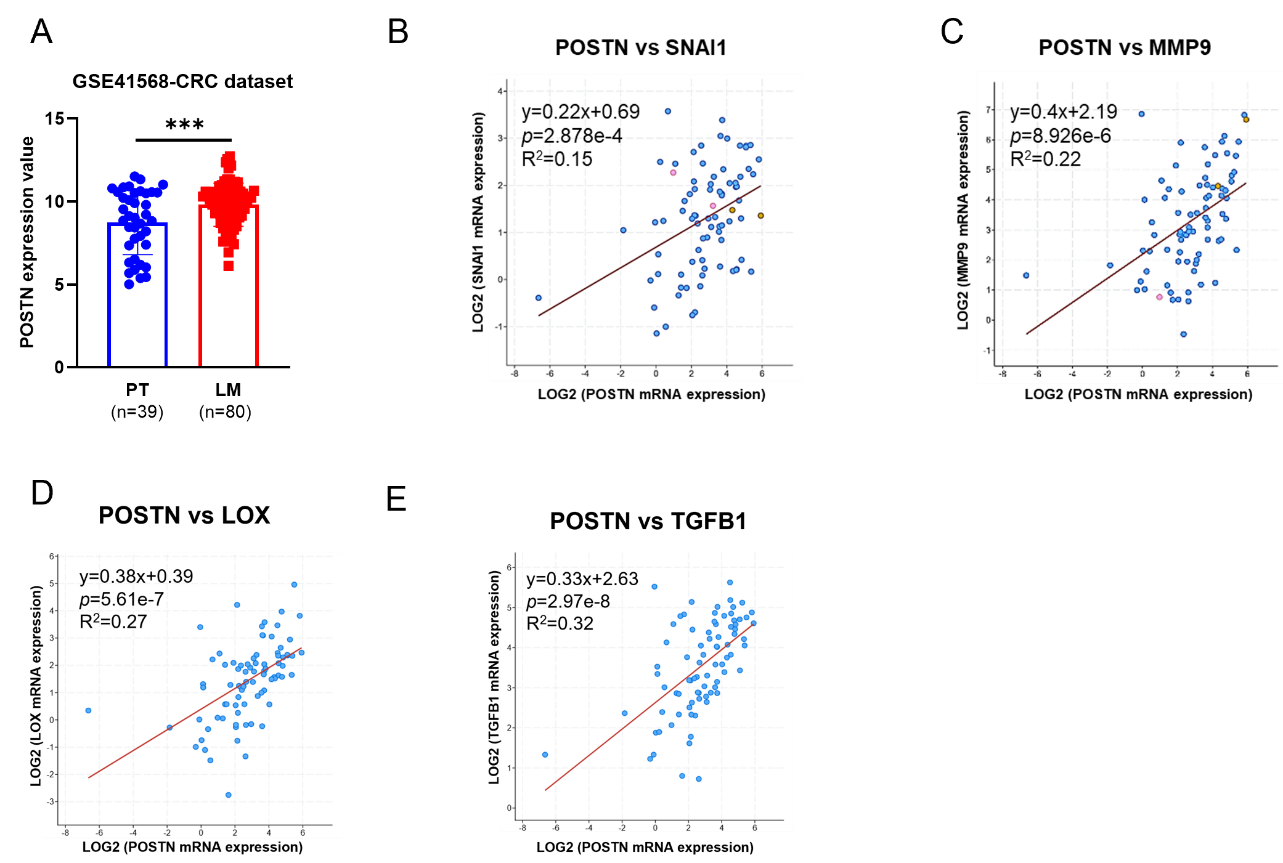


**Figure S1**. A. POSTN expression levels in primary tumors (n = 39, PT) and liver metastases (n = 80, LM) in colon cancer GSE41568 dataset; B-E. Pearson’s correlation analysis of the expression levels of POSTN and SNAI1/MMP9/LOX/TGFB1 in SCLC tumor samples from the cBioPortal database. **P* < 0.05, ***P* ≤ 0.01, ****P* < 0.001, *****P* ≤ 0.0001. Student's t-test. All data were shown as means ± sem.


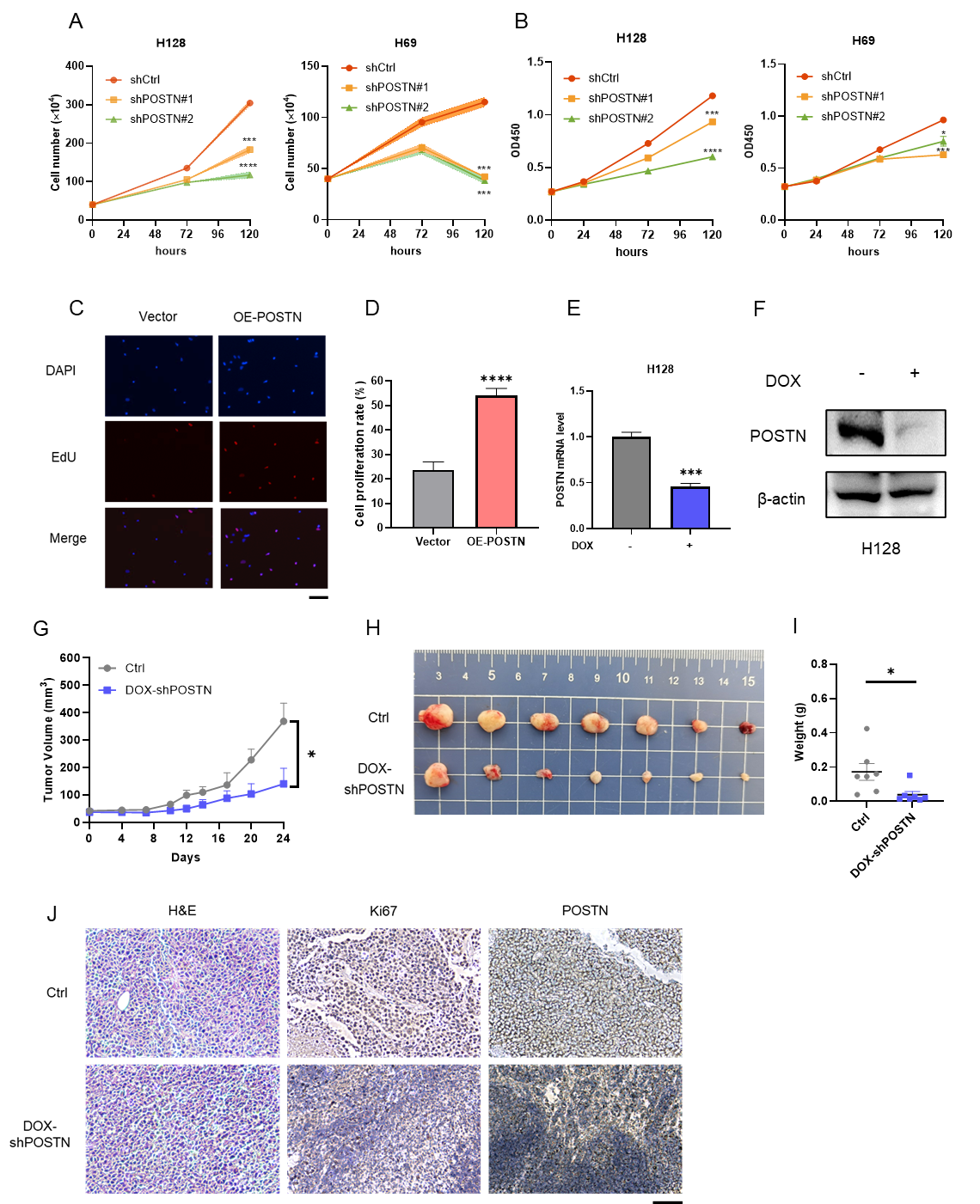


**Figure S2**. A-B. Cell growth determined by cell numbers (A) and CCK8 assay (B) in POSTN-knockdown H128 cells and H69 cells. C. Cell proliferation determined by EdU assay in POSTN-overexpression H446 cells; D. Quantification of cell proliferation ratio; E. The mRNA levels of POSTN in human SCLC cells H128 after DOX induced POSTN knockdown; F. The protein levels of POSTN in H128 in human SCLC cells after DOX induced POSTN knockdown; G. Tumor growth curve of mice injected with SCLC cells in 24-day DOX induction; H. The appearance of the subcutaneous tumor after 24 days of DOX induction; I. The subcutaneous tumor weight after 24 days of DOX induction; J. H&E and IHC staining of Ki67 and POSTN in control group and DOX induced POSTN knockdown group. **P* < 0.05, ***P* ≤ 0.01, ****P* < 0.001, *****P* ≤ 0.0001. n = 3. Difference between two groups was tested by two-sided Student’s t test. All data were shown as means ± sem.


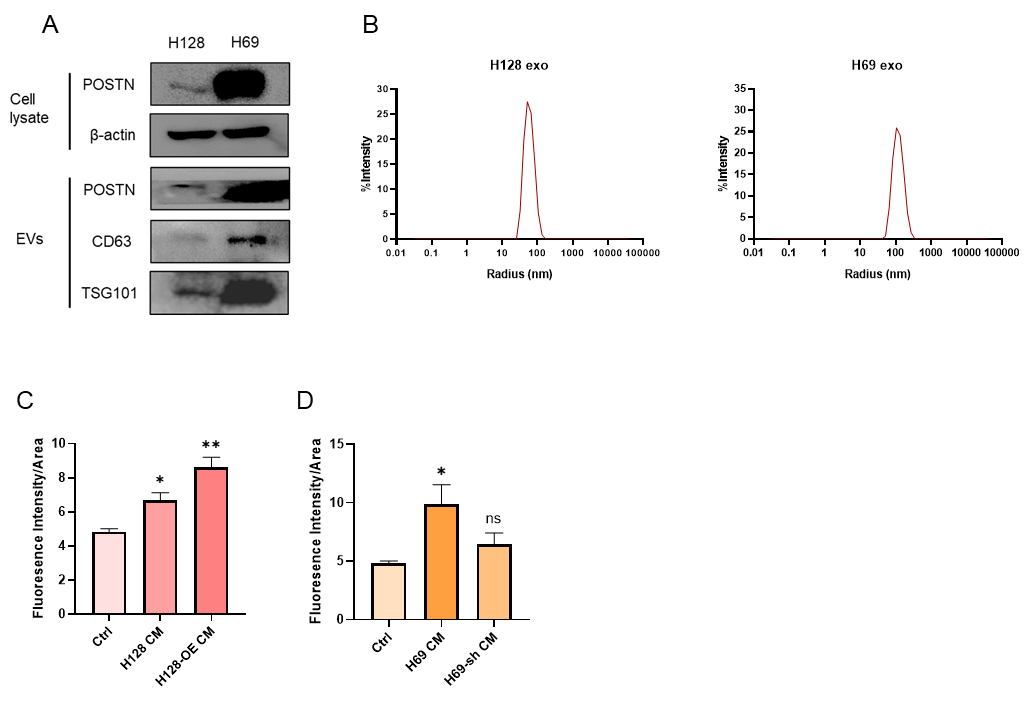


**Figure S3**. A. The protein levels of exosome markers CD63 and TSG101 in SCLC cells exo; B. Size distribution of SCLC cells exo was detected by DLS; C-D. Quantification of α-SMA fluorescence indensity in different SCLC CM treated groups as indicated. **P* < 0.05, ***P* ≤ 0.01, ****P* < 0.001, *****P* ≤ 0.0001. n = 3. Difference between two groups was tested by two-sided Student’s t test. All data were shown as means ± sem.


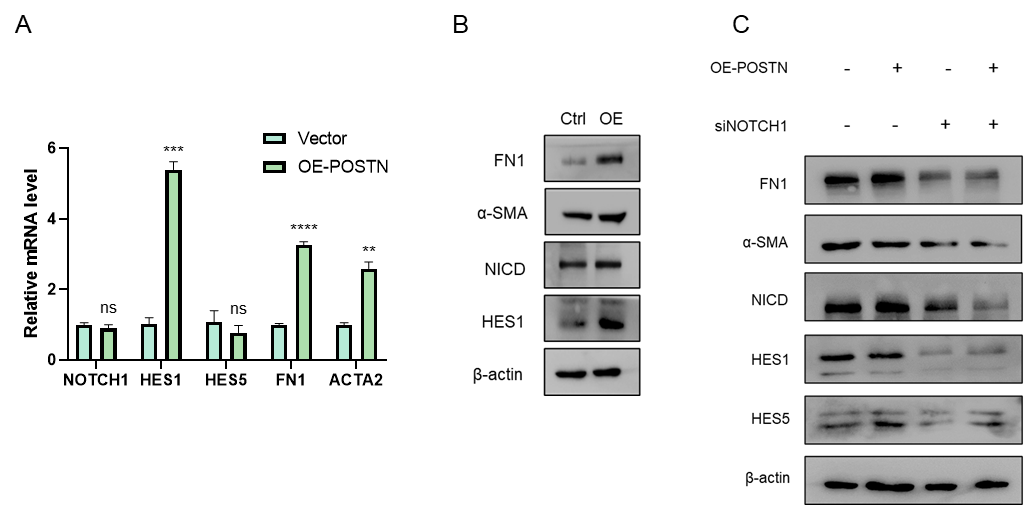


**Figure S4**. A. The mRNA levels of indicated genes in POSTN-overexpressed LX-2 cells; B. The indicated protein levels in POSTN-overexpressed LX-2 cells; C. The indicated protein levels in POSTN-overexpressed LX-2 cells treated with NOTCH1 siRNA as indicated. **P* < 0.05, ***P* ≤ 0.01, ****P* < 0.001, *****P* ≤ 0.0001. n = 3. Difference between two groups was tested by two-sided Student’s t test. All data were shown as means ± sem.


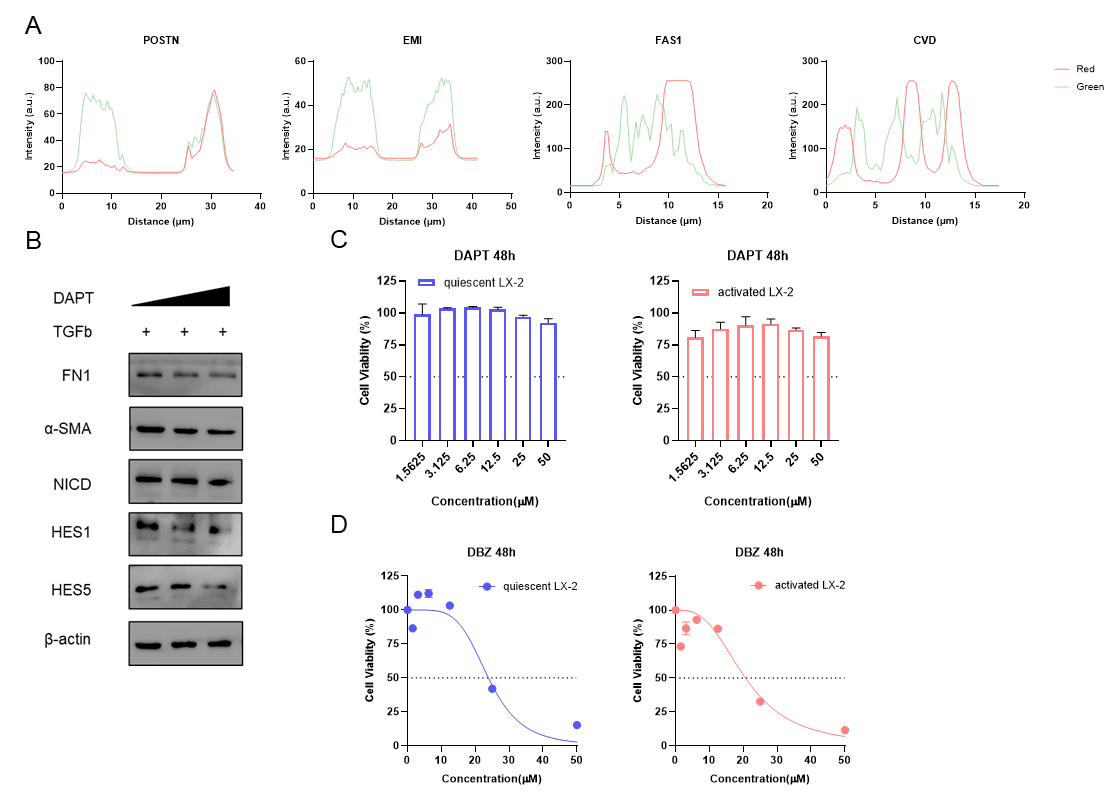


**Figure S5**. A. Quantification of the fluorescence intensity based on Immunofluorescence assay; B. After TGFb stimulation, western blot analysis of fibrosis markers and NOTCH1 pathway protein expression in LX-2 treated with different concentrations of DAPT (0, 20, 50 μM); C. Cell viability of quiesent or activated LX-2 cells treated with DAPT at different concentrations for 48h; D. Cell viability of quiesent or activated LX-2 treated with DBZ at different concentrations for 48h.

**Supplemental Table1-2**

Supplementary Table 1. Quantitative analysis of POSTN expression detected by IHC in human SCLC tissues

| **Age** | **Gender** | **Stage** | **Positive Cells, %** | **Positive Cells Density, number/mm²** | **Mean Density** | **H-Score** | **LOG10 (H-Score)** |
| --- | --- | --- | --- | --- | --- | --- | --- |
| 50 | Male | IA3 | 0.145 | 890 | 0.3813 | 25.47 | 1.4060 |
| 60 | Male | IA2 | 0.1135 | 1177 | 0.5195 | 23.22 | 1.3659 |
| 69 | Male | IA3 | 0.1213 | 710 | 0.3995 | 21.77 | 1.3379 |
| 63 | Male | IA2 | 0.1112 | 634 | 0.4546 | 20.51 | 1.3120 |
| 50 | Male | IA2 | 0.1171 | 686 | 0.3884 | 20.04 | 1.3019 |
| 56 | Male | IB | 0.0801 | 774 | 0.5585 | 16.16 | 1.2084 |
| 63 | Male | IB | 0.079 | 566 | 0.5121 | 16 | 1.2041 |
| 71 | Male | IA2 | 0.0736 | 903 | 0.4988 | 13.62 | 1.1342 |
| 71 | Male | IIB | 0.6722 | 3368 | 0.5623 | 150.22 | 2.1767 |
| 71 | Male | IIB | 0.4446 | 3380 | 0.4505 | 83.32 | 1.9207 |
| 55 | Male | IIB | 0.3895 | 2881 | 0.5759 | 77.78 | 1.8909 |
| 53 | Male | IIB | 0.3213 | 1762 | 0.4657 | 64.14 | 1.8071 |
| 68 | Male | IIA | 0.2578 | 1399 | 0.3716 | 48.27 | 1.6837 |
| 55 | Male | IIB | 0.1441 | 1320 | 0.5846 | 28.68 | 1.4576 |
| 58 | Female | IIB | 0.1271 | 881 | 0.4095 | 22.32 | 1.3487 |
| 60 | Male | IIB | 0.0296 | 270 | 0.4637 | 5.19 | 0.7152 |
| 53 | Male | IIIA | 0.7418 | 3596 | 0.4067 | 144.96 | 2.1612 |
| 61 | Male | IIIA | 0.5594 | 3256 | 0.5128 | 115.77 | 2.0636 |
| 46 | Female | IIIA | 0.3474 | 2767 | 0.6361 | 72.59 | 1.8609 |
| 71 | Male | IIIA | 0.3202 | 1415 | 0.4711 | 67 | 1.8261 |
| 67 | Male | IIIB | 0.1724 | 1179 | 0.3999 | 31.64 | 1.5002 |
| 59 | Male | IIIC | 0.1261 | 360 | 0.4853 | 24.84 | 1.3952 |
| 67 | Male | IIIA | 0.0908 | 504 | 0.4961 | 17.56 | 1.2445 |
| 54 | Male | IIIA | 0.0923 | 1062 | 0.5771 | 17.15 | 1.2343 |

Supplementary Table 2. The sequences of human gene-specific primers

| Gene | Forward Sequence（5'-3'） | Reverse Sequence（5'-3'） |
| --- | --- | --- |
| β-actin | ACTCTTCCAGCCTTCCTTCC | CGTACAGGTCTTTGCGGATG |
| POSTN | CAGCAAACCACCTTCACGGATC | TTAAGGAGGCGCTGAACCATGC |
| FN1 | ACAACACCGAGGTGACTGAGAC | GGACACAACGATGCTTCCTGAG |
| ACTA2 | CTATGCCTCTGGACGCACAACT | CAGATCCAGACGCATGATGGCA |
| COL1A1 | GATTCCCTGGACCTAAAGGTGC | AGCCTCTCCATCTTTGCCAGCA |
| NOTCH1 | GGTGAACTGCTCTGAGGAGATC | GGATTGCAGTCGTCCACGTTGA |
| HES1 | GGAAATGACAGTGAAGCACCTCC | GAAGCGGGTCACCTCGTTCATG |
| HES5 | TCCTGGAGATGGCTGTCAGCTA | CGTGGAGCGTCAGGAACTGCA |
| DLL1 | TGCCTGGATGTGATGAGCAGCA | ACAGCCTGGATAGCGGATACAC |
| DLL4 | CTGCGAGAAGAAAGTGGACAGG | ACAGTCGCTGACGTGGAGTTCA |
| JAG2 | GCTGCTACGACCTGGTCAATGA | AGGTGTAGGCATCGCACTGGAA |
